# Dissemination of multidrug resistant *Acinetobacter baumannii *in various hospitals of Antananarivo Madagascar

**DOI:** 10.1186/1476-0711-9-17

**Published:** 2010-06-30

**Authors:** Tahiry S Andriamanantena, Elisoa Ratsima, Hanitra C Rakotonirina, Frédérique Randrianirina, Lovasoa Ramparany, Jean-François Carod, Vincent Richard, Antoine Talarmin

**Affiliations:** 1 Institut Pasteur de Madagascar, BP 1274, Antananarivo 101, Madagascar; 2 Institut Pasteur de Guadeloupe, Morne Jolivière, BP484, 97183 Les Abymes. Guadeloupe, France

## Abstract

This study reports the dissemination of multidrug-resistant (MDR) OXA-23-producing *Acinetobacter baumannii *clones in hospitals in Antananarivo, Madagascar. A total of 53 carbapenem-resistant *A. baumannii *isolates were obtained from September 2006 to March 2009 in five hospitals. These resistant strains represent 44% of all *A. baumannii *isolates. The double disk synergy test was performed to screen for production of metallo-beta-lactamases. Polymerase chain reaction (PCR) and DNA sequencing were performed for the detection of *bla*(AmpC)*, bla*(OXA-51),*bla*(OXA-23*), bla*(OXA-24*), bla*(IMP), *bla*(VIM). The presence of the insertion sequence IS*Aba1 *relative to *bla*OXA-23 and *bla*OXA-51 was assessed by PCR. Isolates were typed by Rep-PCR. All the isolates were MDR and produced the OXA-23 carbapenemase, which was confirmed by sequencing. PCR analysis for AmpC and OXA-51 gave positive results for all strains studied. No isolates produced metallo-beta-lactamases. In all isolates IS*Aba1 *laid upstream of *bla*OXA-23. The *A. baumannii *isolates were separated into two genotypes; genotype A had a higher prevalence (41 strains) than genotype B (12 strains). Genotype A was present in four hospitals, whilst genotype B had spread in two hospitals. The high frequency of MDR OXA-23-producing *A. baumannii *in various hospitals in Antananarivo is curious since carbapenems are not available in Madagascar, but it emphasises the need for infection control procedures and strict adherence to them to prevent the spread of these resistant organisms in Antananarivo and also the need to control the use of carbapenems in the future.

## 1. Introduction

*Acinetobacter baumannii *is an agent of nosocomial infections, especially pneumonia. It is frequently associated with nosocomial outbreaks worldwide [1,2]. Immunocompromised patients have a high risk of being infected with this organism. *A. baumannii *has become particularly problematic worldwide because of its resistance patterns. Hospital strains of *A. baumannii *are usually multidrug resistant. The problem is aggravated by increasing resistance to broad-spectrum antibiotics including carbapenems, the drugs of choice for hospital-acquired *A*. *baumannii *infections [3-5].

Indeed, carbapenems are generally the last resort in the treatment of lifethreatening infections caused *by Acinetobacter spp*. because they are not affected by most β-lactamases, including extended-spectrum β-lactamases. However their efficacy is increasingly compromised by the emergence of carbapenem-hydrolysing β -lactamase enzymes of Ambler molecular class B (metallo- β-lactamases) and D (oxacillinases) [3,6]. Metalloenzymes are prevalent in East Asia but the OXA-type carbapenemases have emerged as the main mechanism responsible for this resistance worldwide. Recently, the number of OXA-type carbapenemases has increased and they have been divided into eight subgroups of enzymes; four of them have been identified in *A. baumannii*: OXA-23-like (OXA-23, OXA-27 and OXA-49); OXA-24-like (OXA-24, OXA-25, OXA-26, OXA-40 and OXA-72); OXA-58; and OXA-51-like. The last group constitutes a family of chromosomal enzymes typically present in *A. baumannii *[7].

Outbreaks of OXA-23-producing *Acinetobacter *have been reported from various regions of the world [8-13]. During a study on hospital-acquired infections conducted in various surgical units located in two hospitals of Antananarivo, the capital city of Madagascar, *A. baumannii *represented 9.4% of all pathogens isolated. Among them, 44% were resistant to imipenem although carbapenems are not available in Madagascar [14]. We determined the resistance mechanisms of *A. baumannii *to imipenem in Antananarivo and attempted to explain the dissemination of these strains.

## 2. Material and methods

### 2.1. Bacterial isolates

From September 2006 to March 2009, 53 non repetitive clinical isolates of imipenem non-susceptible *A. baumannii*, based on the results of disk diffusion tests, were collected from 53 patients from four hospitals (three public and one private) in Antananarivo, Madagascar. Bacterial isolates were obtained from skin wounds (54.6%), urine (18.9%), respiratory tract secretions (15.1%), blood (5.7%) and other sites (5.7%). Most of the isolates were obtained from patients admitted to the Soavinandriana (HOMI) (45.3%) or Ravoangy Andrianavaolona (HJRA) (39.6%) hospitals. Five control strains obtained at the same time from other patients at two of the hospitals studied and susceptible to carbapenems, with minimum inhibitory concentrations (MICs) of 0.5-2 μg/ml for imipenem were used.

Isolates were identified by conventional techniques. They were kept frozen at -20°C in skimmed milk until further testing.

### 2.2. Antimicrobial susceptibility testing

The disk diffusion method was used to assess susceptibility to the following antimicrobial agents: amikacin; tobramicin; gentamycin; ticarcillin/clavulanic acid; piperacillin/tazobactam; ceftazidime; ampicillin/sulbactam; ciprofloxacin; sulfamethoxazole/trimethoprim, imipenem and colistin. MIC values of imipenem were determined using agar plate dilution. Two-fold serial dilutions of imipenem were added to molten Mueller-Hinton agar base (Oxoid) at a temperature of 45°C. The resulting plates were seeded with 10^4 ^cfu/spot of bacteria by means of a multipoint inoculator and incubated at 37°C for 24 h. *Escherichia coli *ATCC 25922 and *Pseudomonas aeruginosa *ATCC 27853 were used as controls and included in each run. The imipenem concentration range was from 0.5 to 512 μg/ml. MIC values of meropenem were determined by Etest (BioMérieux, Marcy l'Etoile, France) according to the manufacturer's instructions. The meropenem concentration range from 0.02 to 32 μg/ml. Antimicrobial susceptibility was scored using breakpoint criteria shown in table 1 as defined by the Antibiogram Committee of the French Microbiology Society (CASFM) [15], except for ampicilin/sulbactam for which we used the criteria of the Clinical and Laboratory Standards Institute [16].

**Table 1:** Susceptibility pattern of 53 imipenem-non-susceptible *Acinetobacter baumannii *strains to 12 antimicrobial agents

**Antimicrobial**	**Disk Diffusion**							
	**Disc content**	Zone diameters* (mm)		Equivalent MICs breakpoints * (mg/l)		**Results of disk diffusion n (%)**		
		
		S	R	S	R	S	I	R
Amikacin	30 μg	≥17	<15	≤8	>16	25 (47,2)	1 (1,9)	27 (50,9)
Tobramycin	10 μg	≥16	<16	≤4	>4	36 (67,9)	0	17 (32,1)
Gentamycin	15 μg	≥16	<16	≤4	>4	1 (1,9)	0	52 (98,1)
Ampicillin/Sulbactam‡	10 μg/10 μg	≥15	≤11	≤8/4	≥32/16	42 (79.2)	0	11 (20.8)
Ticarcillin/clavulanic acid	75/10 μg	≥22	<18	≤16/2	>64/2	1 (1,9)	0	52 (98,1)
Piperacilin-Tazobactam	75/10 μg	≥19	<14	≤16/4	>64/4	0	1 (1,9)	52 (98,1)
Ceftazidime	30 μg	≥21	<19	≤4	>8	3 (5,7)	0	50 (94,3)
Ciprofloxacin	5 μg	≥22	<19	≤1	>2	1 (1,9)	0	52 (98,1)
Sulfamethoxazole/trimethoprim	23.75/1.25 μg	≥16	<13	≤38/2	>76/4	0	0	53 (100)
Imipenem	10 μg	≥24	<17	≤2	>8	0	4 (7.5)	49 (92.5)
Colistin	50 μg	≥15	<15	≤2	>2	53 (100)	0	0
	MICs			S	R	MIC50 (mg/l) MIC 90 (mg/l)		
Imipenem				≤2	>8	32		32
Meropenem				≤2	>8	≥32		≥32

*: According to the Antibiogram Committee of the French Microbiology Society except for Ampicillin/sulbactam

‡: According to the Clinical and Laboratory Standards Institute

S: susceptible; I: intermediate; R: resistant.

Carbapenem-resistant *A. baumannii *(CRAB) isolates were screened by the modified Hodge test, to evaluate the inactivation of imipenem by carbapenemases, [17].

All strains were tested for the presence of metallo-enzymes using the imipenem-EDTA double-disk synergy test [17].

### 2.3. PCR amplification and sequencing

DNA was extracted from the isolates by boiling five colonies in 250 μl of sterile ultrapure water for 10 minutes, followed by cooling in ice for 10 minutes and centrifugation for 1 min at 14,000 rpm. Supernatants were conserved at -20°C until amplification. Genes coding for class B and D carbapenemases were sought by PCR using Taq polymerase with specific primers. Attempts were made to detect and map the presence of IS*Aba1 *relative to *bla*OXA-23 and *bla*OXA-51 using the primer pairs IS*Aba1*F/OXA-23-seqR (expected size 1456 bp) and IS*Aba1*F/OXA-51-R (expected size 1223 bp), respectively. All primers used are listed in table 2.

**Table 2:** Primers used for the detection of carbapenemase genes.

Name	Sequence	Use	Experimental conditions	Ref
**AmpC**	5'- ACTTACTTCAACTCGCGACG -3' 5'- TAAACACCACATATGTTCCG -3'	^*bla*^ampC Amplification	Classical PCR (annealing temperature 44°C)	[18]
**OXA-51**	5'- ATGAACATTAAAGCACTCTTAC -3' 5'- CTATAAAATACCTAATTGTTCT -3'	^*bla*^OXA-51 Amplification	Classical PCR (annealing temperature 50°C)	[19]
**OXA-23**	5'- GCAAATA*M*AGAATATGT*S*CC -3' 5'- CTC*M*ACCCA*R*CC*R*GTCAACC -3'	^*bla*^OXA-23 Amplification & sequencing	Classical PCR (annealing temperature 58°C)	[20]
**OXA-24**	5'- GGTTAGTTGGCCCCCTTAAA -3' 5'- AGTTGAGCGAAAAGGGGATT -3'	^*bla*^OXA-24 Amplification	Classical PCR (annealing temperature 59°C)	[21]
**VIM**	5'- GTTTGGTCGCATATCGCAAC -3' 5'- CTACTCAACGACTGAGCGATTTGT -3'	^*bla*^VIM Amplification	Classical PCR (annealing temperature 60°C)	[13]
**IMP**	5'- CTACCGCAGCAGAGTCTTTG -3' 5'- AACCAGTTTTGCCTTACCAT -3'	^*bla*^IMP	Classical PCR (annealing temperature 50°C)	[13]
**IsAba-1 F/OXA-23 R**	5'- CACGAATGCAGAAGTTG - 3' 5'-TTAAATAATATTCAGCTGT - 3'	Regulation of OXA-23 by IsAba- 1	Classical PCR (annealing temperature 50°C)	[22]

**REP**	5'-IIIGCGCCGICATCAGGC-3' 5'-ACGTCTTATCAGGCCTAC-3	REP-PCR Amplification	Classical PCR (annealing temperature 40°C)	[23]

The amplified products were observed after electrophoresis on a 1% agarose gel with ethidium bromide staining; purified, amplified products were then sequenced with the dye termination cycle sequencing technique (Genome Express Company, Meylan, France). Searches and alignments for the nucleotide sequences were performed with the BLAST program http://www.ncbi.nlm.nih.gov/BLAST.

### 2.4. REP-PCR

REP-PCR, which uses consensus primers for the REP sequences found in many bacterial chromosomes, was used in the genotyping of *A. baumannii *clones [23,24]. This highly conserved REP sequence is approximately 35 nucleotides long, includes an inverted repeat, and can occur in the genome singly or as multiple adjacent copies. The primer pair REP 1 (5'-IIIGCGCCGICATCAGGC-3') and REP 2 (5'-ACGTCTTATCAGGCCTAC-3') was used to amplify putative REP-like elements in the bacterial DNA. Amplification PCRs were performed as described previously [23,24]. A negative control to detect reagent contamination was included in each PCR, containing all components except the DNA extract, which was replaced by 5 μl of sterile distilled H_2_O. Aliquots (12 μl) of each sample were subjected to electrophoresis in a 1.5% agarose gel. Amplified products were detected by staining with ethidium bromide and photographed with Polaroid type 665 film.

To group isolates for photographic documentation, the REP-PCR fingerprints of strains were exposed to UV light, photographed, and compared by visual inspection. The molecular sizes of fragments generated by electrophoresis were estimated from comparisons with relative molecular mass standards run concurrently. Fingerprints were considered to be highly similar when all visible bands from the two isolates had the same apparent migration distance. Variations in the intensity or shape of bands were not taken into account. The absence of up to two bands from a fingerprint was allowed, when all other visible bands in the fingerprints matched, before isolates were considered to be different by visual inspection [23,24]. Each isolate was run in duplicate. Fingerprint profiles were interpreted with no knowledge of the clinical data.

## 3. Results

According to the results of the antimicrobial susceptibility testing by disk diffusion for 53 imipenem non-susceptible *A. baumannii *isolates, all isolates were resistant to most of the antimicrobials tested. Apart from colistin to which all isolates were susceptible, the highest rates of susceptibility were observed with ampicillin/sulbactam (79.2%; n = 42), tobramycin (67.9%; n = 36) and amikacin (47,2%; n = 25) (Table 1). According to the results of MICs, one isolate was intermediate and all isolates were resistant to imipenem and to meropenem. The MIC of imipenem varied slightly; for 13 isolates it was 16 μg/ml, for 39 isolates it was 32 μg/ml whereas for one strain it was 64 μg/ml. All strains had MICs ≥ 32 μg/ml to meropenem. No synergy was observed using the imipenem-EDTA double-disk synergy test. Using the modified Hodge test, the presence of carbapenemases was detected in 46 (86,8%) of the CRAB consistent with PCR results. Indeed, using PCR and DNA sequencing of the PCR product, all 53 CRAB isolates showed the presence of *bla*OXA-23 and *bla*OXA-51 but none had *bla*OXA-24-like, *bla*OXA-58, *bla*IMP or *bla*VIM. In susceptible isolates, only *bla*OXA-51 was detected.

IS*Aba1 *was detected only upstream of *bla*OXA-23 in all isolates.

**Figure 1:**
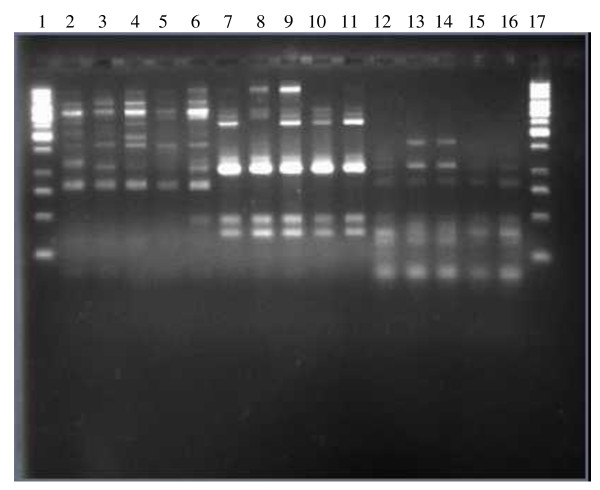
**Representative REP-PCR fingerprints of *A. baumannii *isolates from different human samples, corresponding to the different genotypes**. Lanes 1 and 17, molecular size marker; lanes 2 to 6, genotype A from four hospitals; lanes 7 to 11, genotype B from two hospitals; lanes 12 to 16, five carbapeneme susceptible *A. baumanii *isolates from Antananarivo.

Isolates were analysed by REP-PCR to determine the genomic diversity of CRAB. Two genotypes were observed. The five carbapenem-susceptible control isolates belonged to different genotypes from the CRAB (figure 1). Genotype A (77.4% of isolates; n = 41) was the most prevalent and was found in four hospitals, whilst genotype B (22.6% of isolates; n = 12) had spread in two hospitals.

## 4. Discussion

Our study revealed that OXA-23 carbapenemase was present in *A. baumannii *isolates in Antananarivo. OXA-23-type carbapenemase-producing *A. baumannii *are becoming increasingly widespread, with reports from Europe [25-27], South America [9,13,28], or Asia [29-31]. In 2002, 49 strains of imipenem resistant *A. baumannii *producing the carbapenenase OXA-23 were isolated in South Africa [32]. Many strains of OXA-23 producing *A. baumannii *from the same clone were responsible for an epidemic of nocomial infection from 2005 to 2007 in Tunisia [33].

As in the present study, such isolates usually exhibit resistance to many antimicrobials, creating a serious problem for choice of therapy. In our study, patients with skin infections caused by carbapenem-resistant *A. baumannii *received local treatments and most survived without sequelae. On the contrary, among patients with urinary, pulmonary or blood stream infections who received only antibiotics ineffective on the *A. baumannii *strains isolated (third generation cephalosporins, ciprofloxacin or amoxicillin-clavulanic acid), the rate of mortality was high. Indeed 2 of 3 patients with bloodstream infection, 4 of 8 patients with pulmonary infection and 2 of 10 patients with urinary infection died.

In this study, the OXA-23-producers originated from two clones. According to Rep-PCR patterns, it appears that OXA-23-producing CRAB belonging to two predominant genotypes spread between public and private hospitals in Antananarivo. Different studies have already shown that clones of CRAB may spread in a town [13] or even a country [34]. The occurrence of common Rep-PCR types in OXA-23-producing *A. baumannii *from various hospitals in Antananarivo suggests that dissemination of isolates contributes to the increase in prevalence of CRAB. Dissemination of CRAB in the community could be favoured by poor health facilities and the transfer of infected or colonised patients from a hospital to another. However, this is rarely the case in Antananarivo. Sharing of common healthcare staff is more likely. Indeed staff from public hospitals also works together in the various clinics of Madagascar. This hypothesis has already been evoked to explain the spread of clonal strains between different hospitals [13,35]. If poor sanitary conditions probably explain how these CRAB clones can spread, it does not explain how these clones appeared in Antananarivo. One can suppose that resistant clones were introduced by patients treated in a developed country (hospitalization in Reunion Island is rather frequent for Malagasy patients). However, since carbapenem are not available in Madagascar, resistance to these drugs should not confer any advantage to these clones. Therefore the selection of these clones is probably due to their resistance to most drugs used in Madagascar than to resistance to imipenem.

## Conclusions

Whatever the reason for this spread, the high level of CRAB in the hospitals of Antananarivo is a cause for concern as carbapenems are not available in Madagascar. Thus, the presence of MDR OXA-23 producing *A. baumannii *genotypes emphasises the need for infection control procedures and strict adherence to them to prevent the spread of these resistant organisms in Antananarivo and also the need to control the use of carbapenems in the future.

## Competing interests

The authors declare that they have no competing interests.

## Ethical approval

Not required.

## Authors' contributions

TSA and ER participated in the design of the study, performed the MICs, the genotyping and the characterization of betalactamase, HCR performed the genotyping and the characterization of betalactamase, FR, LR and JFC participated in the collection of the strains and the antimicrobial susceptibility testing, VR participated in the analysis of the results and AT participated in the design of the study and in the analysis of the results. All authors contributed in the writing of the article, read and approved its final version.
